# Occupational Therapy for Parenting: Perspectives of Parents With Physical Disability

**DOI:** 10.1155/2024/4854903

**Published:** 2024-08-16

**Authors:** Anne Honey, Jessica Peterson, Veronica O'Mara, Margaret McGrath

**Affiliations:** ^1^ School of Health Sciences and Centre for Disability Research and Policy The University of Sydney, Camperdown 2006, Australia; ^2^ Sargent College Boston University, Boston, Massachusetts 02215, USA; ^3^ Department of Occupational Science and Occupational Therapy University College Cork, Cork T12 AK54, Ireland

## Abstract

Parenting with a physical disability often brings with it a range of challenges. Occupational therapists are well positioned to support parents to address these challenges, yet occupational therapy research and practice around parenting is relatively scarce. This paper addresses the questions: (1) How should occupational therapists support parenting occupations for people with physical disability? (2) How do parents with physical disability experience occupational therapy? An anonymous survey of 62 parents, primarily mothers, with physical disability about their experiences with parenting challenges and occupational therapy was analysed using both quantitative and qualitative techniques. Parents experienced challenges in engaging in a range of parenting tasks with children over a range of age groups. Parents reported that support was often needed and indicated that occupational therapy could assist them directly with specific parenting goals as well as the more usual biomechanical goals that influence parenting. Yet less than half of participants who received occupational therapy services reported that parenting tasks were addressed, and only one-fifth reported that their goals had been fully met. The data also indicated that the knowledge, skills, and attitudes of occupational therapists with regard to working with parents with disability can be improved. Findings suggest a need to better incorporate parenting occupations in standard occupational therapy training to increase occupational therapists' comfort and competence in working with clients on parenting issues.

## 1. Introduction

Many people with disability parent children. For example, recent estimates indicate that 4.4 million or around 6.7% of parents in the United States have a disability [1]. The International Convention on the Rights of Persons with Disabilities enshrines in Article 23 the right of people with disability to parent and have families.

Around 77% of people with disability report that their main difficulties relate to physical impairments [2]. While parents with physical disability can parent successfully and use creative adaptive strategies to do so, many experience challenges and need or want professional support, which is not always available (e.g., [3–8]).

Occupational therapists are well positioned to take a central role in supporting parenting occupations for people with physical disability, and such work is within their scope of practice [9]. Parenting is one of the most common and meaningful occupational roles for adults, and supporting that role is consistent with the profession's values of occupational justice and strength-based person-centred care that seeks to understand the individual rather than making assumptions based on a diagnosis [10].

Occupational therapists do work with parents with disability. For example, they help parents with physical disability to develop adaptive childcare strategies, prescribe adaptive equipment (e.g., [8, 11–13]), assist with fatigue and pain management in the context of parenting [14, 15], and support the transition to motherhood (e.g., [16, 17]). Yet overall, occupational therapy involvement in supporting parenting remains the exception rather than the rule.

Similarly and relatedly, occupational therapy research and scholarship has tended to neglect parenting as an occupation, particularly for people with disability. Occupational therapy research around parenting has primarily focused on parents caring for children with disability or special needs [18]. A smaller amount of occupational therapy and occupational science literature focuses on understanding general parenting, without a disability focus. These explore, for example, the transitions to and throughout parenthood or the nature of parenting roles and occupations, exploring concepts like co-occupation (e.g., [19–22]). In the authors' previous scoping review of occupational therapy literature that examined the occupations involved in the role of parent [10], 28 of the 104 texts identified focused on parents with disability or health condition, of which 19 related to parents with physical disability. Only one of these was an empirical evaluation of an intervention, though several nonempirical papers described occupational therapy intervention strategies. This review did not represent all occupational therapy and occupational science papers about parenting with physical disability, as papers were excluded if they did not specifically discuss parent occupations, for example, if they focused on parent identity or confidence. Nevertheless, it is clear that the occupational therapy literature, while showing an upward trajectory in terms of the publication of articles about parenting, continues to neglect the occupations of parenting in comparison to those of areas of life such as employment or self-care.

Occupational therapy support for parents with disability is often seen as a specialist area of practice but should not be restricted to specialist services. As a common and critical occupation, occupational therapists working in diverse areas of practice should offer this support just as they would support parents with other valued occupations like self-care and work. Yet research suggests that this is not the case. While no similar data are available for occupational therapists working in physical disability, a 2016 qualitative study found that occupational therapists working in mental health were uncertain about their role in supporting parenting and lacked confidence about their expertise on parenting [23]. As a result, although all of the occupational therapists in this study viewed parenting as a significant part of their clients' lives and were “easily able to identify clients who required support” with parenting (p. 39), none worked with them on parenting-related goals. This lack of confidence is likely to be related to the lack of focus on parenting occupations in occupational therapy curricula, textbooks, and conference proceedings [10, 24].

Little is currently known about the experiences of parents with physical disability around occupational therapy support. Some research has mentioned this issue briefly [7, 8, 25], suggesting that experiences are mixed. However, to the authors' knowledge, no previous research has specifically focused on the experiences of parents with disability with occupational therapy.

This paper is aimed at answering the following research questions: (1) How should occupational therapists support parenting occupations for people with physical disability? and (2) How do parents with physical disability experience occupational therapy?

## 2. Methods

This paper reports the analysis of a cross-sectional survey using both quantitative descriptive methods and interpretive content analysis (ICA) [26]. ICA is a hybrid method which combines qualitative and quantitative techniques, enabling the inductive identification of themes from textual data and their quantification to identify frequency.

The survey was conducted as a needs assessment for part of Author 2's doctoral studies at Boston University. It was therefore deemed by that university's Institutional Review Board not to need IRB approval. Human Research Ethics Committee approval was obtained subsequently from the University of Sydney to analyse and report the anonymous data collected (approval #2023/902).

Authors 1 and 4 are occupational therapy academics, Author 2 is an occupational therapist working in private practice in the area of matrescence (https://www.matrescenceot.com/), and Author 3 is an occupational therapy student. All authors are part of an international collaboration of occupational therapy academics and practitioners interested in researching and promoting occupational therapy support for parents with disability (https://www.ot4parenting.org/).

### 2.1. Sampling and Recruitment

Participants in the survey were parents who self-identified as having a disability and receiving or needing occupational therapy services. A convenience sample was recruited through social media posts on parenting sites and support organisations on Facebook and Instagram. While the survey did not have any exclusion criteria, only two people without physical disability responded. Therefore, to enable a more targeted exploration of the data, they were excluded from this analysis.

### 2.2. Data Collection

Volunteers completed an online anonymous survey via Qualtrics. As no suitable questionnaire existed, the questions were developed, based on gaps identified in the literature, to address the overall aim of the doctoral project, which was to develop an online resource for parents with disability. Questions were designed to cover a breadth of experiences of need for, referral to, and receipt of occupational therapy services. The survey contained both fixed choice and open-ended questions. Items are provided in Supporting Information (available here). Data were collected between November 2022 and September 2023.

### 2.3. Data Analysis

Quantitative data were analysed using descriptive statistics, and chi-square statistics were calculated to explore the significance of apparent relationships. In line with ICA, free text data were inductively coded. Because ICA does not specify a coding technique, constant comparative analysis was used as it is a well-established and systematic technique [27]. Coding was conducted by the first author. First, open coding was conducted, where each response was examined for the concepts it revealed, and each concept was allocated a descriptive name—the code name. Each new piece of data was examined to see whether it fit any existing codes and whether new concepts were evident. Similar codes were then compared, refined, and grouped into broader categories. Qualitative data analysis software, NVivo (QSR International, 2023), was used to manage the coding. All data were reviewed to ensure that they had been thoroughly coded and final frequency counts established.

## 3. Results

A total of 78 people started the survey. Of these, 14 completed only the demographic questions and were therefore excluded from the sample. A further two participants were excluded because they did not report having a physical disability, leaving a sample of 62 participants. Participants reported a wide variety of disability types, the most common being spinal cord injury (*n* = 24), neurological/central nervous system disorders (e.g., stroke, cerebral palsy, and multiple sclerosis; *n* = 15), musculoskeletal disorders (e.g., muscular dystrophy and fibromyalgia; *n* = 15), and connective tissue disorders (e.g., arthritis and Ehlers–Danlos syndrome; *n* = 6). Less frequently mentioned conditions included sensory, heart, and skin conditions. Fifteen participants reported multiple types of physical disability, and three participants also reported a mental health or cognitive condition.  Table 1 further describes the sample. Participants were primarily female, and nearly 2/3 were from the United States. They reported having between one and three children of varying ages, but mostly under 12 years of age.

### 3.1. How Should Occupational Therapy Support Parenting Occupations for People With Physical Disability?

To provide information on how occupational therapy might support parenting, responses to four survey questions were examined. These asked parents to report the most challenging parenting tasks, the most challenging age group to parent, their hopes for occupational therapy, and what they thought an occupational therapist should know about parenting with disability.

#### 3.1.1. Most Challenging Parenting Tasks

**Figure 1:**
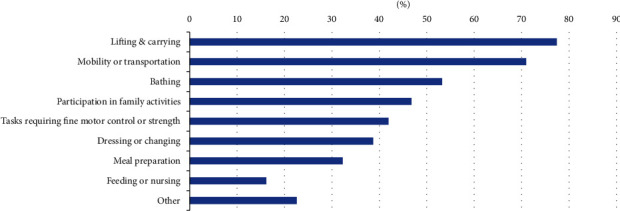
Parenting tasks reported as “most challenging” (*n* = 62).

Parents were able to select multiple parenting tasks as “most challenging,” and they selected between one and nine options (mean = 4). It can be seen in Figure 1 that “lifting and carrying” and “mobility and transportation” were perceived as the most challenging tasks by the greatest percentage of parents. However, most of the task options were seen as “most challenging” by at least one-third of participants. Tasks specified in the “other” category included participating in community activities such as school and playgroups, chasing toddlers, coping with difficult behaviours, shopping and trimming fingernails.

#### 3.1.2. Most Challenging Age Group

**Figure 2:**
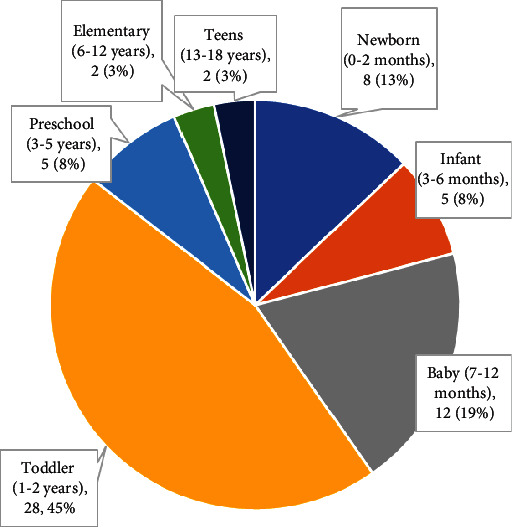
Child age reported as “most challenging” (*n* = 62).

Parents were asked to select the age group they regarded as most challenging and were asked to explain in free text the rationale for their choice. The stages most frequently selected as most challenging (seen in Figure 2) were toddlerhood (*n* = 28; 45%) and babyhood (*n* = 12; 19%). Reasons given usually included the child's increasing physical size and mobility, combined with an inability to understand the parent's limitations or be reasoned with. Parents frequently described issues with handling babies and toddlers as they got larger (*n* = 19; 19%), for example, lifting and carrying them (“I couldn't just scoop him up to leave when he was throwing a fit”) or caring for a resistant or squirming child (e.g., “My son is so strong and won't sit still. I can't change his diaper or his clothes.”) Parents commonly described having trouble “keeping up” with toddlers (*n* = 23; 37%) either because of limited mobility (e.g., “them being faster than me and difficult to reason with”) or limited energy (e.g., “toddler wanted to go go go and I was so fatigued”).

Reasons for the newborn (*n* = 8) and infant (*n* = 5) stages being most challenging also often involved handling issues due to the needs of this age group to be frequently held and carried as well as their perceived fragility. For newborns, a couple of people mentioned issues around being new to parenting and “recovering from labour.”

Nearly three-quarters of parents had children who were older than toddlers, yet these age groups were less frequently seen as most challenging. For parents who chose preschool (*n* = 5), elementary school (*n* = 2), or teens (*n* = 2) as most challenging, the most common reasons were the children's mental health or behavioural issues (*n* = 4), or the parent's difficulty participating in activities (*n* = 3) (e.g., “participating in any sort of event with my children”).

#### 3.1.3. What Participants Wanted From Occupational Therapy

Of 51 people who were referred to occupational therapy, 43 provided interpretable answers to the question of what they hoped to get from it. Seven of the 11 not referred to occupational therapy described how they thought an occupational therapist could help them. Of these 50 participants, 18 (36%) had goals that were interpreted as specific to parenting, including things like “how to fulfill my babies needs despite pain threshold,” “finding equipment to suit my needs as a disabled parent,” and “how to move around while holding the baby.” Fourteen (28%) described physical goals that would be likely to help with parenting as well as other tasks, such as “strength,” “more use of left hand,” and “mobility and dexterity.” Thirteen (26%) described more general goals such as “more independence,” “ADLs,” and “ability to complete tasks.” Six people (12%) indicated that they had no particular hopes or were unsure. As one parent noted, “I did not know what to expect from occupational therapy. After seeing the survey, I think it would be awesome to find an occupational therapist that focuses on parents with disabilities.”

#### 3.1.4. What Occupational Therapists Should Know About Parenting With Disability

Forty-nine participants suggested things they thought occupational therapists needed to know about parenting with a disability. One of the most frequently mentioned themes centred around wanting therapists to take a positive and affirming stance toward them (*n* = 22; 45%). Parents wanted occupational therapists to acknowledge and respect their parental status, accepting that “disabled people have children,” “we can do it,” and “I am not a less effective parent.” They wanted occupational therapists to show this positivity and respect by listening to and trusting parents and by encouraging them: “I need someone to tell me I'm doing a good job.”

Nevertheless, a number of parents wanted occupational therapists to know that parenting with a disability was not easy (*n* = 12; 24%). As one noted, “So much of the job of being a parent is challenging without a disability, so when you add in an additional challenge it can make things feel impossible at times.” Fatigue was a common difficulty: “everything takes twice as much energy.” Other participants mentioned stress: “the emotional toll it takes on a parent with such limited mobility.” Ignorance and environmental barriers were also discussed: “it's challenging when other people don't understand that the set up and lack of equipment is what's preventing me from being independent with a newborn.”

Relatedly, the strongest theme of all was that parents often needed or would benefit from support to parent (*n* = 28; 57%). Some people (*n* = 19) expressed this in a general way: “sometimes we need support,” “find work arounds with specific disabilities,” “we just need better support, modifications, time and patience,” and “we need aids to help.” Others (*n* = 10) wanted occupational therapists to support particular types of activities, such as “bonding activities,” diapering, cooking, or “resourceful ways to incorporate rest.” Two participants wanted occupational therapists to recognise the importance of and facilitate peer support.

Additional advice to occupational therapists included being:

• knowledgeable and realistic (*n* = 6; 12%), for example, “do your research on rare disorders” and “We need actual useful tools that will help us to be better parents and spouses - don't give us exercises that are useless”
• holistic (*n* = 4; 8%), for example, “it's more than diaper changes” and “anything that helps us be involved in our kids' activities and lives is useful”
• proactive (*n* = 4; 8%), for example, “Spend time with your patient watching the activities, don't just ask what's hard”
• flexible and inclusive (*n* = 2; 4%), for example, “I couldn't attend any mom and baby or mom and tots workshops because no one had disability in mind”

### 3.2. How Do Parents With Disability Experience Occupational Therapy?

Of the 51 participants who were referred to occupational therapy, 43 reported receiving occupational therapy services; however, six participants did not answer any further questions in the survey. Experiences of occupational therapy for parenting were therefore reported by 37 parents (60% of the total sample). Of these parents, 21 (57%) received the occupational therapy service as an inpatient in a hospital, 21 (57%) in an outpatient setting, 19 (51%) in their own home, 1 in a group setting, and 2 via telehealth. Nineteen participants (51%) received occupational therapy services in more than one of these settings. We examined their responses to questions about the parenting tasks addressed by the occupational therapist, the extent to which their goals were met by occupational therapy, and the perceived knowledge and attitudes of the occupational therapist.

#### 3.2.1. Parenting Tasks Addressed

**Figure 3:**
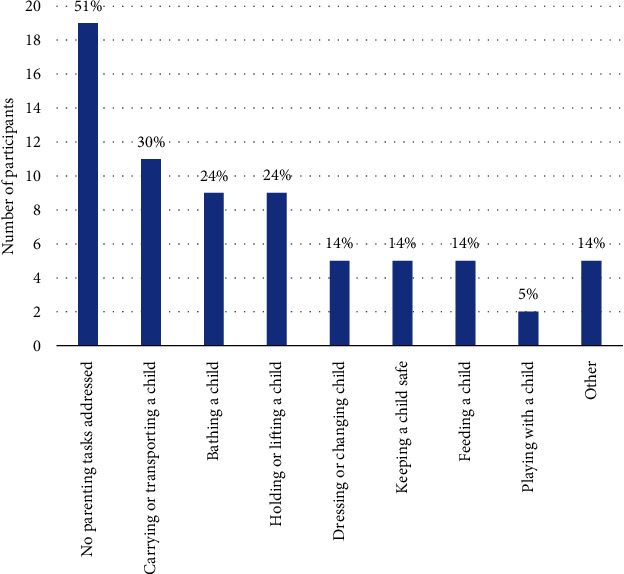
Parenting tasks addressed in occupational therapy (*n* = 37).

As can be seen in Figure 3, for more than half participants (*n* = 19; 51%), no parenting tasks were addressed by occupational therapy. Other participants reported between one and six parenting tasks being addressed (mean = 1.4). The most frequently addressed parenting tasks were carrying, transporting, holding, lifting, and bathing. The “other” category consisted of sitting postures, being safe while walking with the child, attending to the child at night, sensory processing, coping with difficult behaviours, and communication.

#### 3.2.2. Meeting of Goals

**Figure 4:**
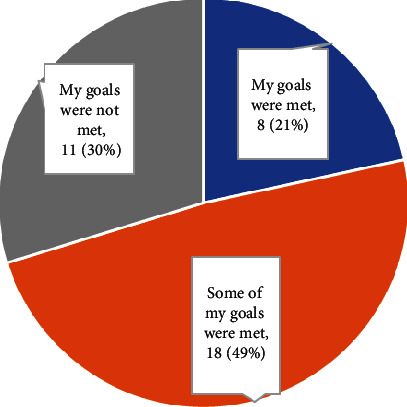
Personal goals met by occupational therapy (*n* = 37).

Figure 4 shows that 70% of participants believed that occupational therapy had met at least some of their personal goals, but only around one-fifth reported that their goals were fully met.

Participants who reported that occupational therapy addressed parenting in some way were more likely to report that their goals were met (83.3% vs. 57.9%); however, a chi-square test of independence showed no significant relationship between having parenting addressed and meeting goals, *X*^2^ (1, *N* = 37) = 2.17, *p* = 0.14.

#### 3.2.3. Occupational Therapist Knowledge and Attitudes

**Figure 5:**
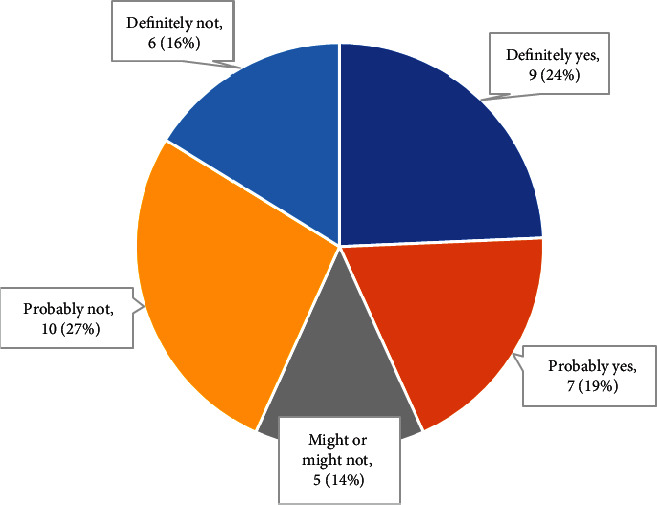
Did you feel that the occupational therapist was knowledgeable about your disability? (*n* = 37).

It can be seen from Figure 5 that, of the participants who received occupational therapy, less than half considered the occupational therapist to be definitely or even probably “knowledgeable about your disability.”

Further, more than half of responding participants (19/34; 56%), when asked, said that during the course of receiving occupational therapy, they had experienced “lack of professional knowledge of how to address your goals” and more than half (19/34; 56%) reported that they had experienced bias, discrimination, or generalisation of disability. Only 12 participants (35%) reported none of these experiences.

While chi-square tests of independence indicated no statistically significant relationships, two interesting trends were observed. More participants whose occupational therapist addressed parenting considered the occupational therapist to be knowledgeable about their disability than those whose occupational therapist did not address parenting (55.6% compared to 31.6%: *X*^2^(1, *N* = 37) = 2.18, *p* = 0.14). On the other hand, people whose occupational therapist addressed parenting were also more likely to report that their occupational therapist lacked professional knowledge of how to address their goals (63.2% compared to 38.9%: *X*^2^(1, *N* = 37) = 2.18, *p* = 0.14 and 2.86, *p* = 0.09).

## 4. Discussion

This exploratory study is the first to examine the views of parents with disability of occupational therapy services. It adds to the small body of literature about occupational therapy and parenting and provides an understanding of the need for and experiences of occupational therapy among parents with physical disability.

The findings suggest that parents experience challenges with a range of parenting tasks. The tasks parents find most challenging are broadly in line with previous findings. For example, Wint et al. [8] described parents with physical disability needing to find adaptations for bathing, feeding, and lifting and carrying children. The most frequent responses—lifting/carrying and mobility/transportation—also align with the interventions most frequently reported by occupational therapists as being addressed [11]. However, the fact that only one task of the options given—“feeding or nursing”—was selected as most challenging by less than 20% of parents indicates that parents found a wide range of tasks challenging. Further, the high number of people who rated “participation in family activities” as most challenging emphasises that parents may benefit from supports beyond the traditional biomechanical aspects of diapering, bathing, feeding, and carrying. It also supports research that identifies many community barriers to parenting, such as the inaccessibility of schools, public transport, parks, and other recreational facilities and the negative attitudes and perceptions of other people [28].

It is unsurprising that the most frequently cited “most challenging age” for children was toddlerhood and that the reasons usually cited were the child's increasing physical size and mobility combined with the developmental limitation on their ability to be reasoned with. This is something that occupational therapists seem to understand, as a scoping review of occupational therapy literature on the doing of parenting found that 70% of texts related to children aged 2 years old or younger [10]. Research on the experiences of parents with disability has also tended to focus more on parenting younger children [28]. Yet a significant minority of parents in our study found parenting older children more challenging. The evolving nature of parenting through a child's developmental stages means that occupational therapists need to consider parenting occupations for parents of children at each developmental stage. For researchers, this also indicates a need to ensure that studies of parenting with disability go beyond the early years and consider the experience and needs of people parenting children through middle childhood and beyond.

Parents in this study wanted occupational therapists to understand that they needed support with parenting, but many expressed very general hopes for occupational therapy or were not sure of what it could provide. This suggests an overall lack of knowledge about occupational therapy and the possibilities it offers, which is likely to result in many parents either not actively seeking occupational therapy or being unable to articulate to an occupational therapist what they want or need in terms of their parenting occupations. This is in line with the generally low profile of occupational therapy in the area of parenting [10, 11]. Occupational therapists, therefore, need to be proactive with addressing parenting needs by enquiring about and assessing parenting needs rather than relying on parents to ask.

It is concerning that less than half of those who saw an occupational therapist received any support with parenting. While these parents were likely referred to occupational therapy for issues that were not parent specific, the importance of parenting as an occupation and the many challenges identified in this and other studies (e.g., [3–8]) suggest that this would have been an important opportunity for parenting issues to be identified and addressed. Further, nearly a third of parents who received occupational therapy identified that their goals were not met, suggesting that occupational therapists need to better attend to understanding the needs of their clients.

The high proportion of parents reporting inadequate knowledge on the part of the occupational therapist reflects research with occupational therapists who provide services to people with physical disability, a majority of whom rated training in parenting assessment and interventions as a very high (31%) or high (43%) priority for them [11]. This is also understandable in the context of the lack of parenting training in current occupational therapy curricula [24]. It is also congruent with the views of parents with disability about professional services generally, which are often seen as lacking an understanding of the issues associated with parenting with disability (e.g., [29–32]). Interestingly, there was a trend for occupational therapists who addressed parenting to be more often seen as knowledgeable about disability but also as lacking knowledge about how to address the person's goals. Failure to reach statistical significance may be a result of the small sample size, so these relationships should be examined in future studies. Perhaps only more experienced occupational therapists took on parenting, but occupational therapists were more competent in addressing nonparenting goals rather than parenting ones.

Finally, the results emphasise the importance of occupational therapists taking a positive and affirming stance toward parents with disability. There is clearly room for improvement in this area with more than half of participants reporting the experience of bias, discrimination, or generalisation of disability. This supports previous multidisciplinary literature in which many parents with disability have reported being stigmatised by health professionals who assume that they are incompetent or that their children are disadvantaged (e.g., [32, 33]).

### 4.1. Limitations

The study has several limitations. As with any survey relying on a convenience sample of volunteers, the sample may not be representative of the wider populations of parents with disability. Although our study was open to all genders, the gender balance of respondents means that the findings primarily represent the experiences of mothers. Importantly, participants may be more engaged with or passionate about issues of parenting with disability than other parents. For example, they were engaged with the parenting social media groups where the study was advertised, chose to participate in the study, and, unlike 18% of those who started the survey, continued after the demographics questions. Further, the sample size was relatively small, increasing the potential impact of this bias and possibly leading to failure to identify associations from the data. The survey itself was constructed for the specific purpose of the professional doctorate, limiting the data available to answer research questions more broadly relevant to occupational therapists.

## 5. Conclusions

Notwithstanding the limitations, this study provides important preliminary information on the experience of parents with disability around occupational therapy services. Findings suggest a need for additional training for occupational therapists, preferably in their preregistration degrees, to increase their comfort and competence in working with clients on parenting issues. They also highlight the need for additional research: both quantitative research to verify the findings and better estimate the frequency of different experiences of occupational therapy and qualitative research to dig deeper and develop a more nuanced understanding of parents' experiences. Given parenting is one of the most valued and widespread of all adult occupations, all occupational therapists working with people with disability of parenting age should be well prepared to address parenting issues with their clients.

## Figures and Tables

**Table 1:** Participant characteristics.

**Characteristic**	**# (** **n** = 62**)**	**%**
Age	Mean = 37; sd = 6.5; range = 23–61	
Gender identity		
Female	55	88.7%
Male	6	9.7%
Nonbinary	1	1.6%
Type of physical disability		
Spinal cord injury	24	
Musculoskeletal disorders	15	
Neurological/CNS disorders	15	
Connective tissue disorders	6	
Heart conditions	3	
Sensory condition	2	
Other physical conditions	8	
Number of physical disability types reported		
1	47	75.8%
2	5	8.1%
3	5	8.1%
> 3	5	8.1%
Country of residence		
United States	40	64.5
United Kingdom	8	12.9
Canada	7	11.3%
Australia	3	4.8%
Other (South Africa, Norway, Kenya, and India)	4	6.5%
Number of children		
1	33	53.2%
2	21	33.9%
3	7	11.3%
Missing	1	1.6%
Age/s of child/ren?		
Less than 12 months	11	
1–2 years	18	
3–5 years	20	
6–12 years	30	
13–18 years	13	
> 18 years	5	
Referred to OT services while a parent	51	82.3%
Received OT services while a parent	37	59.7%

## Data Availability

The survey data used to support the findings of this study may be available via application to the authors.

## Supporting Information

Supporting Information Additional supporting information can be found online in the Supporting Information section. Parenting survey–questions asked: This document provides a list of the questions asked in the survey. This is to provide a full context for the analysis.
