# Supplementary material for: Occupational Therapy for Parenting: Perspectives of Parents With Physical Disability
Source: Occup Ther Int. 2024 Aug 16;2024:4854903. doi: 10.1155/2024/4854903 (PMC11343624; doi:10.1155/2024/4854903)
Supplement: Supporting Information — Additional supporting information can be found online in the Supporting Information section. Parenting survey–questions asked: This document provides a list of the questions asked in the survey. This is to provide a full context for the analysis. [file 4854903.f1.docx]

Appendix 1: Survey questions and response options (questions not analysed for this paper are shaded grey).

| **Question** | **Type of question** | **Response options (selected choice questions)** |
| --- | --- | --- |
| Age | Free text |  |
| Gender Identity | Selected choice | Male; Female; Non-binary/third gender; Prefer not to say |
| Number of children | Free text |  |
| Disability diagnosis or type of disability | Free text |  |
| What country do you currently live in? | Free text |  |
| What is/are the age(s) of your child(ren)? | Free text |  |
| How long have you been parenting with a disability? | Free text |  |
| What types of parenting tasks have you found to be the most challenging? Choose any that apply | Selected choice | Lifting and carrying;  Tasks requiring fine motor control or strength;  Dressing or changing;  Bathing;  Mobility or transportation;  Ability to participate in family activities;  Feeding or nursing;  Meal preparation;  Other, please describe below. |
| (If other) What types of parenting tasks have you found to be the most challenging? | Free text |  |
| In your experience, which one of the marked age groups above has been the most challenging to parent with a disability? | Selected choice | Newborn (0-2 months);  Infant (3-6 months);  Baby (7-12 months);  Toddler (1-2 years);  Preschool (3-5 years);  Elementary (6-12 years);  Teens (13-18 years);  Adults (18+ years) |
| Please describe was the most challenging for you about parenting the above age group? | Free text |  |
| Is there anything about parenting with a disability that you would like occupational therapy practitioners to know? | Free text |  |
| Where did you learn about occupational therapy? | Free text |  |
| Have you ever been referred to occupational therapy? | Selected choice | No  Yes |
| Who referred you to occupational therapy? | Selected choice | Hospital physician  Primary care provider  Physical therapist  Orthopedist  Other, please describe |
| (If other): Who referred you to occupational therapy? | Free text |  |
| Did you end up receiving OT services? | Selected choice | No  Yes |
| (If yes) When you were referred, what did you hope to get out of occupational therapy? | Free text |  |
| (If no) What was the reason that you did not receive OT services? | Free text |  |
| Describe any ways you think occupational therapy could benefit you | Free text |  |
| Did you need to ask for a referral? | Selected choice | Yes, the referral was given according to my request;  No, a provider suggested the referral  Other, please describe |
| (If other) Did you need to ask for a referral? | Free text |  |
| (If yes) Did you need to ask multiple providers before being given the referral? | Free text |  |
| How long did it take to receive services after the initial referral? | Free text |  |
| In what setting have you received occupational therapy? Please select all that apply. | Selected choice | In my home;  In an outpatient clinic;  In an inpatient hospital setting;  In a community or group setting;  Virtual/telehealth;  Other (please describe) |
| (If other) In what setting have you received occupational therapy? | Free text |  |
| What was the focus of the visits? Please select all that apply. | Selected choice | Exercise;  Completing tasks of daily living;  Education;  Learning a new skill;  Recommendations for assistive technology;  Home or environment modification;  Adaptive driving;  Other, please describe. |
| What was the focus of the visits? | Free text |  |
| What parenting tasks were addressed by the occupational therapist? Please select all that apply. - | Selected choice | Dressing or changing a child  Bathing a child  Carrying or transporting a child  Holding or lifting a child  Keeping a child safe  Playing with a child  Feeding a child  Other, please describe |
| (If other) What parenting tasks were addressed by the occupational therapist? | Free text |  |
| To what extent were your personal goals met by occupational therapy? | Selected choice | My goals were met;  Some of my goals were met;  My goals were not met |
| Did you feel that the occupational therapist was knowledgeable about your disability? | Selected choice | Definitely not  Probably not  Might or might not  Probably yes  Definitely yes |
| During the course of receiving occupational therapy, did you experience any of the following? | Selected choice | Bias (supporting or opposing a particular person in an unfair way);  Discrimination (the unfair or prejudicial treatment of people and groups based on personal characteristics);  Lack of professional knowledge of how to address your goals;  Generalisation of disability. |
